# An ERA-CRISPR/Cas12a Method for Highly Sensitive Detection of Human Adenovirus Type 55

**DOI:** 10.3390/diagnostics15212725

**Published:** 2025-10-27

**Authors:** Letian Zhang, Zhenghan Luo, Taiwu Wang, Yifang Han, Fuqiang Ye, Chunhui Wang, Yue Chen, Jinhai Zhang

**Affiliations:** 1 Center for Disease Prevention and Control of Eastern Theater of Chinese PLA, Nanjing 210002, China; 18115142863@163.com (L.Z.); IrvingHan@126.com (Z.L.); wangtaiwu@hotmail.com (T.W.); imhanyifang@163.com (Y.H.); nkuyfq@163.com (F.Y.); 13912966353@139.com (C.W.); 2 Department of Microbiology, Faculty of Naval Medicine, Naval Medical University, Shanghai 200433, China; 3 Center for Disease Prevention and Control of Southern Theatre of Chinese PLA, Guangzhou 510507, China

**Keywords:** HAdV55, ERA, CRISPR-Cas12a, nucleic acid detection method

## Abstract

**Background/Objectives:** Human adenovirus 55 (HAdV55) is a notable pathogen causing community-acquired pneumonia; outbreaks occur frequently in military camps, hospitals, and schools, thereby posing a threat to public health security. This study aimed to develop a method for detecting HAdV55 nucleic acid by targeting the conserved region of the Hexon gene. The sequence was amplified using enzymatic recombination isothermal amplification (ERA) technology, in conjunction with CRISPR-Cas12a technology, to enhance the amplification signal. **Methods:** Optimized primer and crRNA sequences were selected through ERA isothermal amplification testing. The ERA-CRISPR/Cas12a detection method was completed within 30 min at a constant temperature of 42 °C. **Results:** Sensitivity was assessed by detecting standard plasmids and live strains at various dilution concentrations. The detection limits were determined to be 9 copies/reaction for standard plasmids and 2.5 copies/reaction for cultured HAdV55 strains. Specificity tests were conducted on positive samples for five common respiratory pathogens and five other adenovirus subtypes, all of which showed no cross-reactivity. **Conclusions:** A rapid ERA-CRISPR/Cas12a nucleic acid detection method for HAdV55 has been successfully developed, demonstrating high sensitivity and specificity without the need for expensive or complex instruments. This method holds promise for on-site pathogen screening and detection.

## 1. Introduction

Human adenovirus (HAdV) is a double-stranded DNA virus classified under the genus *Mastadenovirus* within the family *Adenoviridae*. It comprises seven serological groups (A–G) and at least 116 subtypes [1,2]. HAdV is widely acknowledged as a common etiological agent of acute respiratory disease (ARD) [3,4]. HAdV55, a member of serotype B, is a recombinant virus primarily consisting of genetic segments from adenovirus type 14, with hypervariable regions originating from adenovirus type 11 [5,6,7]. First isolated in China in 2006, HAdV55 is predominantly transmitted via respiratory droplets and has high transmission capability and pathogenicity [8]. It can induce symptoms of acute respiratory infections, such as fever, cough, headache, sore throat, bronchitis, and pneumonia, which may escalate to severe pneumonia or even result in death [9]. Environments such as crowded communities, military barracks, and schools are typical settings for HAdV55 infections [10]. Owing to the absence of specific antibodies against HAdV55 in the population, this virus can easily trigger outbreaks, potentially leading to severe public health consequences [11,12,13]. Currently, no specific therapeutic drugs are available for adenoviruses. Thus, developing a rapid on-site detection method with high sensitivity, strong specificity, and ease of operation is crucial for early diagnosis and effective epidemic prevention and control.

With the advancement of molecular biology, nucleic acid detection technology has become integral to pathogen diagnosis. Traditional nucleic acid detection methods, such as conventional PCR and quantitative real-time PCR, involve complex procedures and extended detection times. These methods require thermal cycling instruments and rigorous operating conditions and are limited by factors such as equipment availability, cost, power supply, and space. Consequently, these constraints hinder their use in resource-limited settings or for rapid on-site detection. As a result, isothermal nucleic acid amplification techniques have attracted considerable attention from researchers [14]. Common isothermal amplification methods include loop-mediated isothermal amplification (LAMP), recombinase polymerase amplification (RPA), and recombinase-aided amplification (RAA).

Enzymatic Recombinase Amplification (ERA) is a novel isothermal amplification technique capable of specifically amplifying trace amounts of target DNA fragments, which is considered an upgraded version of RPA and RAA [15]. The principle of ERA is similar to that of RPA and RAA reactions, all of which utilize the DNA-dependent ATPase activity of the UvsX protein. In the presence of the single-stranded DNA-binding gp32 protein, energy generated through ATP hydrolysis is used to displace homologous double-stranded DNA fragments and form displacement loop structures. The key distinction lies in the source of recombinase employed in each technology: RPA utilizes recombinase derived from T4 bacteriophage; RAA employs recombinases from bacteria and fungi; and ERA utilizes recombinases originating from bacteria, viruses, and bacteriophages [15,16]. To further reduce the reaction temperature, ERA technology employs in vitro combinatorial screening or amino acid mutagenesis screening of low-temperature phage-derived proteins such as gp32, UvsX, UvsY, DNA polymerase, and creatine kinase, enabling efficient amplification reactions to proceed at lower temperatures. ERA facilitates the exponential amplification of target gene sequences within 20 min at temperatures ranging from 25 °C to 42 °C, thus eliminating the need for complex temperature control equipment and stringent laboratory conditions associated with conventional nucleic acid amplification techniques [17,18,19]. The application of ERA technology in pathogen nucleic acid detection demonstrates excellent detection performance, showing comparable detection time, specificity and sensitivity to RPA and RAA technologies [16]. Theoretically, it can operate stably under conditions closer to room temperature. However, as a newly developed technique, current research remains limited, and further experiments are needed to explore the potential for advancements in the detection performance of ERA technology.

Clustered regularly interspaced short palindromic repeats (CRISPRs) and CRISPR-associated (Cas) protein systems, identified as adaptive immune systems in bacteria and archaea, effectively protect against the invasion of foreign genetic material [20]. These systems capture segments of the genetic material of the invader and integrate them into the CRISPR locus within the host genome. Upon reinvasion by foreign genetic material, CRISPR RNA (CrRNA) is synthesized to serve as a guide. The CrRNA specifically recognizes complementary gene sequences, forms a complex with Cas proteins, and cleaves the exogenous genetic material. In 2018, researchers discovered that the Cas12a protein, while performing specific cleavage (cis-cleavage), also possesses the ability to nonspecifically cleave nontargeted single-stranded DNA (trans-cleavage) [21,22]. This characteristic enables the CRISPR/Cas12a system to be utilized in nucleic acid detection, where the introduction of reporter molecules into the reaction system facilitates signal amplification. The Cas12a protein forms a complex with crRNA to recognize target sequences complementary to the crRNA. Based on this mechanism, an ssDNA reporter modified with a fluorophore can be designed. When the target sequence is present in the reaction system, the ssDNA reporter is non-specifically cleaved, releasing the fluorophore and allowing rapid detection of fluorescence signals. When the concentration of the target gene in the sample is low, introducing the CRISPR/Cas12a system can amplify the detection signal, thereby enhancing detection sensitivity and specificity [23]. Furthermore, owing to the similarity in reaction conditions, isothermal nucleic acid amplification techniques are often combined with the CRISPR/Cas12a system to establish detection methods. Researchers have integrated the CRISPR/Cas12a system with techniques such as LAMP, RPA, RAA and ERA for the detection of pathogens such as hepatitis C virus, methicillin-resistant *Staphylococcus aureus* and African swine fever virus [24,25,26,27,28].

**Figure 1:**
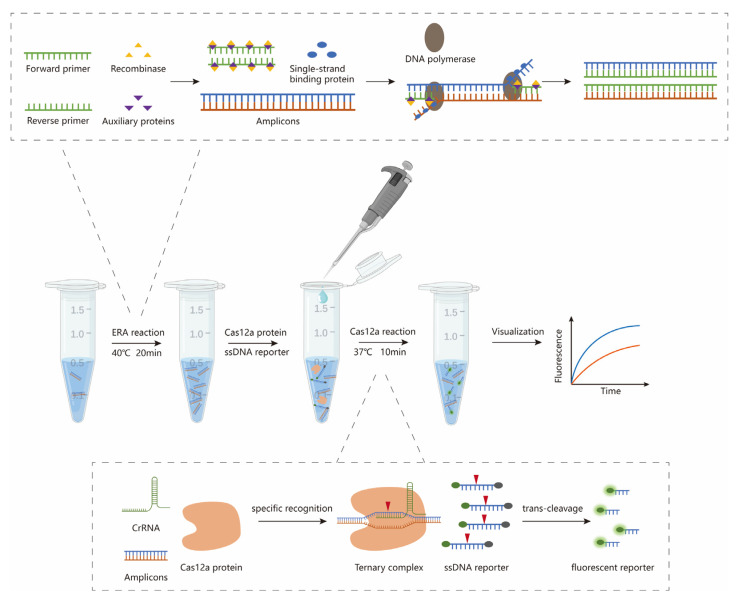
The workflow of the overall process for HAdV55 detection is based on the ERA-CRISPR/Cas12a assay and can be completed within 30 min.

In this study, ERA technology was combined with the CRISPR/Cas12a system to develop a highly sensitive, specific and rapid nucleic acid detection method for human adenovirus type 55 (Figure 1). The ERA-CRISPR/Cas12a technology was first employed for the establishment of an HAdV55 detection method, exploring the feasibility of applying ERA technology for the development of point-of-care testing approaches.

## 2. Materials and Methods

### 2.1. Biological Materials

The following strains were maintained in our laboratory: human adenovirus types 55, 3, 7, 11, 14, and 21 (HAdV55, HAdV3, HAdV7, HAdV11, HAdV14, and HAdV21); influenza A virus (IAV); influenza B virus (IVB); human parainfluenza virus (HPIV); respiratory syncytial virus (RSV); and *Mycoplasma pneumoniae* (MP). The nucleic acid of severe acute respiratory syndrome coronavirus-2 (SARS-CoV-2) was extracted from the inactivated nasopharyngeal swabs of infected individuals. All the pathogen-related experiments were conducted in biosafety cabinets within the corresponding biosafety laboratories. A risk assessment was conducted for each experiment.

### 2.2. Reagents and Instruments

*Bam*HI restriction endonuclease (Takara), pUC57 vector (GenScript Biotechnology Co., Ltd., Nanjing, China), Express Virus RNA Rapid Extraction Kit (Sidagene, Tokyo, Japan, NR202), HAdV55 nucleic acid detection kit (Beijing Kinghawk Pharmaceutical Co., Ltd., Beijing, China), Basic Universal ERA detection kit (Syntegon Gene Co., Ltd., Waiblingen, Germany), and Cas12a protease (Shenzhen Yizhi Biotechnology Co., Ltd., Shenzhen, China) were used.

A fluorescence quantitative PCR instrument (Bio-Rad CFX96, Hercules, CA, USA), a Qubit 4.0 fluorescence spectrophotometer (Thermo Fisher, Waltham, MA, USA), and a Fluorescence Thermal Amplification Instrument GS8 (Syntegon Gene) were used.

### 2.3. Preparation of Positive Control

**Figure 2:**
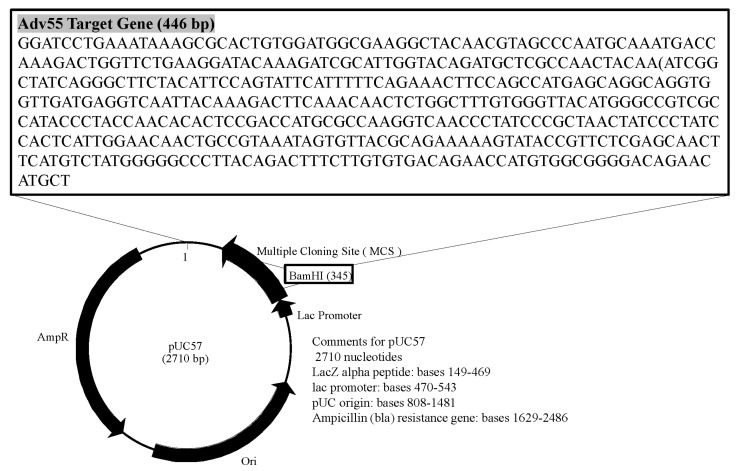
Schematic diagram of the HAdV55 positive control plasmid construct. The HAdV55 positive control plasmid clone was constructed by inserting the Hexon gene sequence of HAdV55 into the pUC57 vector. *Bam*HI was used to linearize the plasmid for inserting the Hexon gene sequence of HAdV55.

The Hexon gene sequence of HAdV55 was obtained from GenBank and aligned using MEGA7 to identify a conserved fragment. The target sequence of the Hexon gene was synthesized by Tsingke Biotechnology Co., Ltd. (Beijing, China). Following the linearization of the pUC57 vector with *Bam*HI, the conserved fragment, measuring 446 base pairs, was ligated into the pUC57 vector and subsequently transformed into *E. coli DH5α*. Recombinant pUC57 plasmid DNA containing the Hexon gene was extracted using a Plasmid Small Extraction Kit (Tiangen, Beijing, China). The resulting recombinant plasmid was verified through Sanger sequencing (Figure 2).

The plasmid concentration (350 ng/μL) was quantified on a Qubit 4.0 fluorometer. On the basis of the formula: concentration (copies·μL^−1^) = (6.02 × 10^23^) × (Con.·ng/μL × 10^−9^)/(length of the plasmid (bp) × 660), the copy number concentration was calculated as 1 × 10^11^ copies/μL. By a 10-fold gradient dilution ratio, positive control plasmid standards with concentrations ranging from 1 × 10^2^ copies/μL to 1 × 10^7^ copies/μL were obtained.

### 2.4. Standard Curve

**Figure 3:**
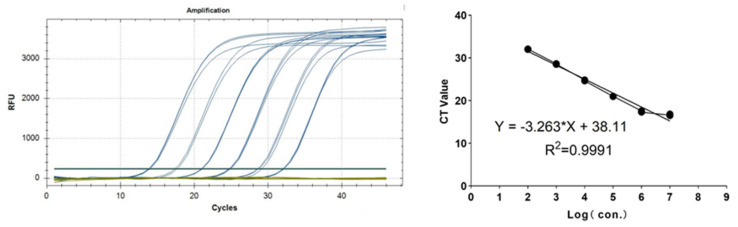
Amplification curves (X-axis: cycle; Y-axis: fluorescence value) and standard curves of the plasmid standards for each plasmid standard with concentrations ranging from 1 × 10^7^ copies/µL to 1 × 10^2^ copies/µL. The Ct values and the logarithm of the initial template from 10-fold dilutions are associated with a coefficient of determination (R^2^) = 0.9991.

It was simultaneously amplified using the HAdV55 detection kit to establish a linear relationship between Ct/fluorescence signal and the logarithm of the initial template, thereby enabling absolute quantification of the unknown samples. Serially diluted positive controls served as templates for real-time PCR following the HAdV55 detection kit protocol; each concentration was run in triplicate. The log_10_ (initial concentration) was plotted against the Ct to generate a standard curve (Figure 3). The regression equation was Y = −3.494X + 38.88, with R^2^ = 0.9991, confirming excellent linearity and reliable serial dilution of the plasmid standard; this curve was subsequently used to quantify the viral copy numbers in the clinical samples.

### 2.5. Design of Primers, Probes and CrRNAs

Probes and primers were designed and evaluated using Primer Premier 5.0, considering their length, GC content, and amplicon size. The optimal length for the ERA primers is between 30 and 35 base pairs (bp). Primers that are too short may impair recombinase activity and decrease detection sensitivity, whereas excessively long primers can reduce amplification efficiency. It is preferable for the 5′ end of ERA primers to start with a cytosine, as this facilitates the recombination of amplified fragments. Additionally, consecutive guanines (more than three) at the 5′ end should be avoided. The 3′ end should ideally consist of cytosine and guanine to promote stable polymerase binding and enhance primer amplification performance. During primer design, palindromic sequences and repetitive structures should be minimized, whereas sequences prone to forming hairpin structures must be avoided to reduce primer dimer formation. Furthermore, a GC content that is excessively high (>60%) or low (<40%) in the primer sequence is detrimental to ERA.

On the basis of the Hexon gene of human adenovirus type 55 obtained from GenBank, target sequences for Cas12a were designed. Using the MUSCLE algorithm in MEGA7, relatively conserved sequences were identified through multiple sequence alignment. The length of the crRNA matching the target sequence is typically 20–23 base pairs, while the crRNA sequence should avoid the formation of hairpin structures and dimers. Within these conserved regions, Cas12a protospacer adjacent motifs (5′-TTTV) were screened to design guide RNA sequences (gRNAs) complementary to the target sites. CrRNAs, which include both gRNAs and conserved stem‒loop structures, were constructed for subsequent Cas12a detection experiments.

One probe (HAdV55BP), six forward primers (F1–F6), six reverse primers (R1–R6), and two CRISPR RNAs (CrRNA1 and CrRNA2) were selected, as detailed in Table 1. All oligonucleotides were synthesized by Tsingke Biotechnology Co., Ltd.

### 2.6. Optimization of CrRNA and ERA Primer–Probe Sets

A plasmid standard at a concentration of 300 copies/μL was amplified using an ERA fluorescence kit. The reaction mixture (50 μL) consisted of 20 μL of rehydration buffer, 2.1 μL of each primer (10 μM), 0.6 μL of HAdV55BP probe (10 μM), 22.2 μL of nuclease-free water, 2 μL of activator, 1 μL of template, and a lyophilized core enzyme. Amplification was conducted in a GS8 fluorimeter at 40–42 °C for 20 min, with fluorescence values from the FAM channel collected every 30 s. A positive result was defined as a fluorescence increase exceeding 300 and the presence of a clear amplification curve, whereas a negative result was indicated by a flat fluorescence trace with no significant increase.

Following the ERA, 1.2 μL of Cas12a (10 μM), 1 μL of CrRNA (10 μM), and 0.5 μL of fluorescent reporter (10 μM) were added to the reaction. CRISPR detection was then performed at 37 °C for 10 min with continuous fluorescence monitoring. The presence of HAdV55 nucleic acid in the test sample was determined on the basis of the resulting curve.

For optimal CrRNA selection, CrRNA1 and CrRNA2 were paired with R1 and each forward primer (F1–F6), and the CrRNA yielding the highest amplification efficiency was selected. The optimal reverse primer was identified by fixing HAdV55BP and F1 and testing with R1–R6. The best forward primer was determined by fixing the selected reverse primer and HAdV55BP and testing with F1–F6. The final combination (HAdV55BP plus the optimal primers) was validated using nuclease-free water as a negative control.

### 2.7. Nucleic Acid Extraction

Viral RNA was extracted using the Express Virus RNA Rapid Extraction Kit according to the manufacturer’s instructions. A total of 150 microliters from each clinical sample was transferred into a 1.5 mL microcentrifuge tube, followed by the addition of 120 µL of RNA LB1. The mixture was immediately inverted five times and incubated for 10 s. Subsequently, 500 µL of BB2 RNA was added, and the tube was inverted five times. The lysate was then loaded onto a spin column and centrifuged at 13,000× *g* for 30 s. The flow-through was discarded, and the membrane was washed in accordance with the kit protocol. Finally, the RNA was eluted in 50 µL of nuclease-free water and stored at −80 °C until further analysis.

### 2.8. Sensitivity Assays

Plasmid-Based Assay: Serial dilutions of a positive control plasmid, ranging from 9 × 10^5^ to 9 × 10^1^ copies/μL, including concentrations of 45 and 9 copies/μL, were evaluated using the optimized primer–probe set in the ERA-CRISPR/Cas12a system. The lowest concentration that consistently resulted in amplification was used to define the limit of detection (LOD) for plasmid DNA.

Live Virus Assay: Cultured HAdV55 was quantified using a standard curve and subsequently diluted to concentrations ranging from 1 × 10^7^ to 1 × 10^5^, including 5 × 10^3^, 50, 2.5, and 1.25 copies/μL. Each dilution was tested with the ERA-CRISPR/Cas12a system using optimal primers and CrRNA to determine the LOD for the infectious virus.

### 2.9. Specificity Assays

Respiratory pathogens with infection sites or clinical symptoms similar to those of HAdV-55, which could potentially cause cross-reactions, were selected for testing. Nucleic acids from respiratory pathogens with overlapping tropisms or clinical presentations—such as IAV, IBV, HPIV, RSV, MP, and SARS-CoV-2—as well as heterotypic human adenoviruses (HAdV-3, HAdV-7, HAdV-11, HAdV-14, and HAdV-21) were extracted and analyzed using the optimized primer–probe set and CrRNA.

## 3. Results

### 3.1. Development of the ERA-CRISPR/Cas12a Method for HAdV55 Detection

**Figure 4:**
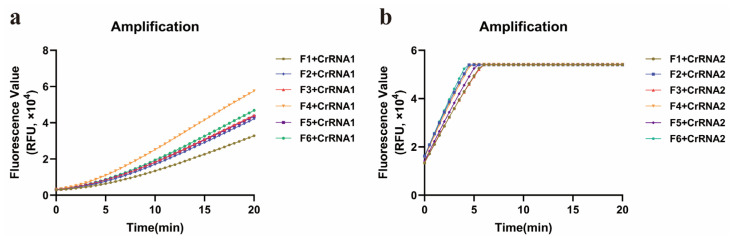
Performance comparison of CrRNAs. (**a**) Amplification curves of CrRNA1 and six forward primers (F1–F6). (**b**) Amplification curves of CrRNA2 and six forward primers (F1–F6).

To perform the ERA, the reverse primer R1 was combined with six forward primers (F1, F2, F3, F4, F5, and F6). ERA-CRISPR/Cas12a reactions involving either CrRNA1 or CrRNA2 were tested with various primer combinations. Compared with that of CrRNA1, the amplification curve of CrRNA2 reached its maximum fluorescence value of approximately 5 × 10^4^ within 5 min. The amplification efficiency of CrRNA2 consistently increased (Figure 4). Consequently, CrRNA2 was selected for subsequent amplification.

### 3.2. Selection of the Optimal ERA Primer–Probe Set

**Figure 5:**
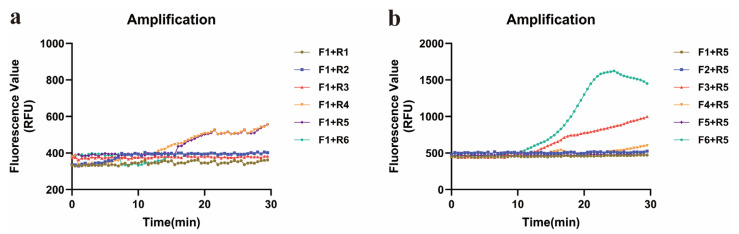
Performance comparison of the forward primers and reverse primers. (**a**) Amplification curves of F1 and six reverse primers (R1–R6). (**b**) Amplification curves of R5 and six forward primers (F1–F6).

HAdV55BP and the forward primer F1 were fixed initially. Six reverse primers were individually paired with F1 and evaluated using ERA-CRISPR/Cas12a assays (Figure 5a). After the most effective reverse primer was identified, HAdV55BP and this primer were held constant, and six different forward primers were tested sequentially (Figure 5b). On the basis of the criteria of maximal fluorescence gain and the earliest onset of amplification, the primer pair F6R5 was selected. Finally, when the reaction was conducted with HAdV55BP and the optimized primer set, the nuclease-free water negative control consistently yielded negative results.

### 3.3. Sensitivity of the ERA-CRISPR/Cas12a Assay

**Figure 6:**
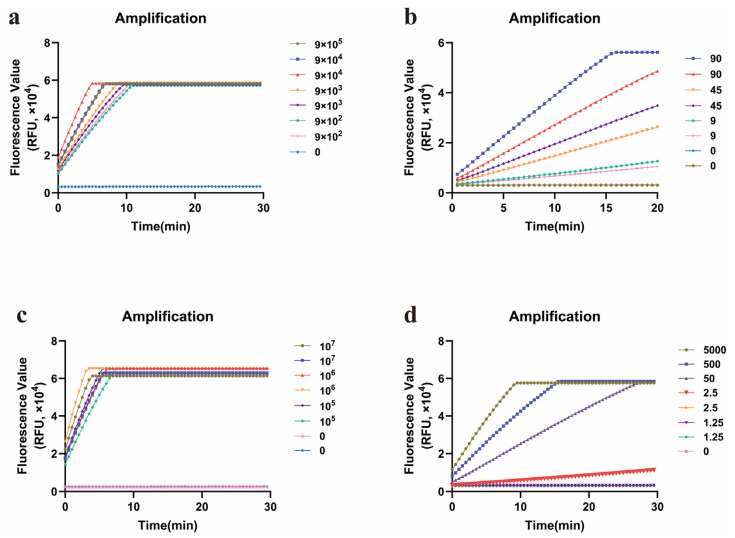
Evaluation of the sensitivity of the HAdV55 ERA-CRISPR/Cas12a assay. (**a**) Amplification curves of HAdV55 for each plasmid standard with concentrations ranging from 9 × 10^5^ copies/µL to 9 × 10^2^ copies/µL. (**b**) Amplification curves of HAdV55 for each plasmid standard at concentrations of 90, 45 and 9 copies/µL. (**c**) Amplification curves of HAdV55 for each cultured HAdV55 strain at concentrations ranging from 10^7^ copies/µL to 10^5^ copies/µL. (**d**) Amplification curves of HAdV55 for each cultured HAdV55 strain at concentrations of 5000, 50, 2.5 and 1.25 copies/µL.

Using the optimal primer set and probe identified through the aforementioned screening process, ERA-CRISPR/Cas12a amplification reactions were conducted on both the positive standard plasmid and the live virus strain across a series of dilutions. For the positive standard plasmid (Figure 6a,b), the optimized primer–probe set achieved a LOD of 9 copies/reaction. In the case of the live HAdV55 virus (Figure 6c,d), the LOD was determined to be 2.5 copies/reaction.

### 3.4. Specificity Assay

**Figure 7:**
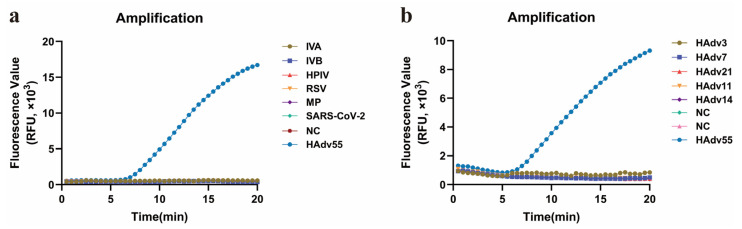
Evaluation of the specificity of the HAdV55 ERA-CRISPR/Cas12a assay by subtypes of HAdV and pathogens that caused respiratory infections. (**a**) Amplification curves representing samples positive for HAdV55. The negative samples were IAV, IBV, HPIV, RSV, MP, SARS-CoV-2 and the negative control. (**b**) Amplification curves representing samples positive for HAdV55. The negative samples were HAdV3, HAdV7, HAdV21, HAdV11, HAdV14 and the negative control.

To evaluate the specificity of the ERA-CRISPR/Cas12a assay, we tested a panel of respiratory pathogens that either colonized the same anatomical sites or produced clinical syndromes indistinguishable from those associated with HAdV55 infection. All non-HAdV55 targets yielded negative results (Figure 7a). Similarly, when representative strains of human adenovirus subtypes other than HAdV55 were tested, the fluorescence remained at background levels (Figure 7b). These findings collectively demonstrate that the assay is highly specific for HAdV55.

## 4. Discussion

In this study, ERA technology was integrated with the CRISPR/Cas12a system to establish a rapid nucleic acid detection method for HAdV55, termed the ERA-CRISPR/Cas12a combined assay. The ERA-CRISPR/Cas12a nucleic acid detection method exhibits exceptional sensitivity, with a detection limit of 2.5 copies/reaction in a 50 μL reaction system. Since the amplification reaction system possesses reverse transcriptase activity, enabling the detection of not only viral genomic DNA but also RNA within the virus. Therefore, the sensitivity of viral isolates is higher than that of plasmids. Lu et al. developed a quantitative fluorescence PCR method for detecting human adenovirus Group C, achieving a reaction time of one hour [29]. Lei et al. devised a LAMP detection method for HAdV7, HAdV14, and HAdV55, with a detection limit of 10 copies/μL for HAdV55 [30]. The method presented in this study surpasses existing nucleic acid detection methods for HAdV55 in terms of sensitivity. Owing to the absence of HAdV55-positive clinical samples, live virus strain samples were used to validate the detection performance of the assay. The development of typing and identification methods for various subtypes of HAdV has been a significant focus and challenge for researchers. Ziros et al. developed a LAMP detection method targeting HAdV40 and HAdV41, enabling visual observation of results after 60 min of isothermal amplification at 69 °C [31]. Qiu et al. introduced a probe-based real-time fluorescence quantitative PCR detection method capable of simultaneously detecting HAdV2, HAdV3, and HAdV7 in a single tube [32]. Wang et al. designed a dual real-time fluorescent RAA nucleic acid detection method for HAdV3 and HAdV7 by targeting the Hexon gene [33]. Through a comparison with RPA, RAA, RT-qPCR, and LAMP techniques for the detection of HAdV in terms of sensitivity and detection time, it can be concluded that the ERA-CRISPR/Cas12a system demonstrates outstanding detection performance and holds broad research prospects (Table 2).

In this study, various adenovirus subtypes that are common respiratory pathogens and may cross-react with HAdV55 were selected. Using the established detection method, no cross-reactions were observed with other adenovirus subtypes, such as HAdV11 and HAdV14, or with common respiratory pathogens, such as influenza A and SARS-CoV-2, confirming the high specificity of the method. HAdV55 is a recombinant virus derived from HAdV11 and HAdV14. The Hexon gene of HAdV55 shows a close evolutionary relationship with that of HAdV11, while its other protein genes exhibit greater similarity to those of HAdV14 [34]. Previously, many detection methods struggled to differentiate between HAdV55, HAdV11, and HAdV14 or lacked validation with relevant viral strains. Given the excellent specificity demonstrated by the primers and CrRNA designed in this study, it is feasible to further develop a multiplex nucleic acid detection method capable of identifying and detecting different HAdV subtypes. Owing to the flexibility in primer and crRNA design, the strategy employed in establishing this detection method is applicable to other HAdV subtypes. If new HAdV subtypes emerge in the future, it will be essential to promptly develop rapid, on-site detection methods with high sensitivity and specificity.

The primary limitation of this method is the requirement for an uncapping step during the detection process to introduce the Cas12a enzyme, which increases the risk of nucleic acid aerosol contamination. Future advancements could focus on developing an integrated detection kit to improve both accuracy and user-friendliness. Additionally, incorporating lateral flow strip technology might eliminate the need for instrumental readouts, enabling visual interpretation of results and making the method more suitable for rapid, on-site testing. Currently, there are relevant studies that combine the CRISPR/Cas system with lateral flow technology to establish detection methods, which show promising application prospects in the field of rapid on-site testing [35,36].

Furthermore, CRISPR-Cas12 detection technology utilizing fluorescent reporting systems can significantly reduce detection time, as fluorescent signals are detectable within one minute of reaction initiation. Notably, the increase in fluorescence intensity is independent of viral load and instead relies on the trans-cleavage efficiency of the Cas12-CrRNA complex. In clinical applications, fluorescence signals can be measured using portable handheld fluorometers or smartphone-based fluorescence detectors. Samacoits et al. developed a cost-effective smartphone-based detection device integrated with machine learning-driven software to analyze fluorescence signals generated by CRISPR/Cas12a reactions [37].

In recent years, emerging technologies based on fluorescent biosensors, oligonucleotide sensors, colorimetric biosensors, and electrochemical biosensors have introduced innovative approaches for pathogen detection. For example, compared with quantitative fluorescent PCR methods, electrochemical biosensors have been used to establish nucleic acid detection methods for influenza B virus, offering greater detection efficiency, making them more suitable for rapid diagnostic scenarios [38]. Moreover, existing studies have applied electrochemical, surface-enhanced Raman scattering (SERS)-based, and colorimetric biosensors for detecting viral particles in food samples [39]. While each sensing technology has distinct advantages and limitations, biosensor technology has demonstrated substantial potential for further development.

The optimization of viral nucleic acid extraction protocols is a crucial and challenging aspect in the development of rapid, on-site nucleic acid detection methods. Bae et al. proposed a technique employing rotating blades and magnetic beads for the swift extraction of viral nucleic acids from samples, achieving viral lysis and nucleic acid absorption in under 60 s. The subsequent washing and elution steps were completed within 5 min, thereby significantly enhancing the efficiency of viral nucleic acid extraction and rendering it more suitable for rapid on-site testing [40]. Future research efforts should prioritize the optimization of nucleic acid extraction methods to increase the efficiency and accuracy of detection methods.

## 5. Conclusions

In summary, the ERA-CRISPR/Cas12a nucleic acid detection method developed in this study eliminates the need for large precision instruments and offers advantages such as simplicity of operation, short detection time, high sensitivity, and strong specificity. Although the method established in this research does not fully meet the requirements for field detection, it identifies directions for future improvements during the investigative process. Future efforts may focus on refining nucleic acid extraction protocols, optimizing operational procedures—such as developing a single-tube detection system to minimize contamination risks from repeated lid openings—and adopting novel result interpretation strategies (e.g., lateral flow dipstick or portable fluorescence analyzers) to meet the demands of rapid on-site testing.

## Figures and Tables

**Table 1:** Primers and probes for the ERA.

Name	Sequence 5′-3′
HAdV55BP	GAAGTTTCTGAAAAATGAATACATGCGA(FAM-dT)(THF)(BHQ-dT) TTGTATCCTTCTG(C3-SPACER)
F1	GCCAACTACAACATCGGCTATCAGGGCTTC
F2	CCAACTACAACATCGGCTATCAGGGCTTCT
F3	TACAACATCGGCTATCAGGGCTTCTACATT
F4	ACAACATCGGCTATCAGGGCTTCTACATTC
F5	CAACATCGGCTATCAGGGCTTCTACATTCC
F6	AACATCGGCTATCAGGGCTTCTACATTCCA
R1	TAATTGACCTCATCAACCACCTGCCTGCTC
R2	AAGTCTTTGTAATTGACCTCATCAACCACC
R3	GAAGTCTTTGTAATTGACCTCATCAACCAC
R4	TGAAGTCTTTGTAATTGACCTCATCAACCA
R5	TTGAAGTCTTTGTAATTGACCTCATCAACC
R6	GGCCTTGAAGTCTTTGTAATTGACCTCATC
CrRNA1	UAAUUUCUACUAAGUGUAGAUAUUUUUCAGAAACUUCCAGC
CrRNA2	UAAUUUCUACUAAGUGUAGAUUGAAAAAUGAAUACAUGCGA

**Table 2:** Comparative performance of different nucleic acid detection methods in detecting human adenovirus.

Technique	Detection Time	Reaction Temperature	Sensitivity (Copies/Reaction)	Pathogen	Transducing Method
RT-qPCR	>1 h	Temperature-controlled cycle	5	HAdVs (serotypes 1, 2, 5 and 6)	Fluorescence
ERA-CRISPR/Cas12a	30 min	37~42 °C	2.5	HAdV55 (in this study)	Fluorescence
RPA	25 min	37 °C	14	HAdVs (serotypes 3, 7, 21, and 55)	Lateral flow
RAA	1 h	37~42 °C	18	HAdVs (serotypes 3 and 7)	Fluo-rescence
LAMP	1 h	69 °C	10	HAdVs (serotypes 7, 14, and 55)	Colorimetric

## Data Availability

The original contributions presented in this study are included in the article. Further inquiries can be directed to the corresponding author.
